# Flip-Flop HSV-BAC: bacterial artificial chromosome based system for rapid generation of recombinant herpes simplex virus vectors using two independent site-specific recombinases

**DOI:** 10.1186/1472-6750-6-40

**Published:** 2006-09-22

**Authors:** Toshihiko Kuroda, Robert L Martuza, Tomoki Todo, Samuel D Rabkin

**Affiliations:** 1 Molecular Neurosurgery Laboratory, Department of Neurosurgery, Massachusetts General Hospital/Harvard Medical School, 185 Cambridge St., CPZN-3800, Boston, MA 02114, USA; 2 Present address: Department of Neurosurgery, The University of Tokyo, 7-3-1 Hongo, Bunkyo-ku, Tokyo 113–8655, Japan

## Abstract

**Background:**

Oncolytic herpes simplex virus (HSV) vectors that specifically replicate in and kill tumor cells sparing normal cells are a promising cancer therapy. Traditionally, recombinant HSV vectors have been generated through homologous recombination between the HSV genome and a recombination plasmid, which usually requires laborious screening or selection and can take several months. Recent advances in bacterial artificial chromosome (BAC) technology have enabled cloning of the whole HSV genome as a BAC plasmid and subsequent manipulation in *E. coli*. Thus, we sought a method to generate recombinant oncolytic HSV vectors more easily and quickly using BAC technology.

**Results:**

We have developed an HSV-BAC system, termed the Flip-Flop HSV-BAC system, for the rapid generation of oncolytic HSV vectors. This system has the following features: (*i*) two site-specific recombinases, Cre and FLPe, are used sequentially to integrate desired sequences and to excise the BAC sequences, respectively; and (*ii*) the size of the HSV-BAC-insert genome exceeds the packaging limit of HSV so only correctly recombined virus grows efficiently. We applied this to the construction of an HSV-BAC plasmid that can be used for the generation of transcriptionally-targeted HSV vectors. BAC sequences were recombined into the UL39 gene of HSV ICP4-deletion mutant d120 to generate M24-BAC virus, from which HSV-BAC plasmid pM24-BAC was isolated. An ICP4 expression cassette driven by an exogenous promoter was re-introduced to pM24-BAC by Cre-mediated recombination and nearly pure preparations of recombinant virus were obtained typically in two weeks. Insertion of the ICP4 coding sequence alone did not restore viral replication and was only minimally better than an ICP4-null construct, whereas insertion of a CMVIE promoter-ICP4 transgene (bM24-CMV) efficiently drove viral replication. The levels of bM24-CMV replication in tumor cells varied considerably compared to *hr*R3 (UL39 mutant).

**Conclusion:**

Our Flip-Flop HSV-BAC system enables rapid generation of HSV vectors carrying transgene inserts. By introducing a tumor-specific-promoter-driven ICP4 cassette into pM24-BAC using this system, one should be able to generate transcriptionally-targeted oncolytic HSV vectors. We believe this system will greatly facilitate the screening of a plethora of clinically useful tumor-specific promoters in the context of oncolytic HSV vectors.

## Background

Oncolytic virus therapy is a new modality of cancer treatment, which has shown promising results in clinical trials [1]. Oncolytic viruses are designed to selectively replicate in and kill cancer cells without harming normal tissue. Since reporting the successful treatment of a brain tumor model by a *tk*-mutant herpes simplex virus (HSV) vector in 1991 [2], we have focused on the development of increasingly efficacious and safe, replication-competent oncolytic HSV vectors. For the generation of new oncolytic vectors, insertion of exogenous genes or promoter sequences is often performed to enhance anti-tumor activity or tumor-selectivity of the vector. An example of the former is insertion of an interleukin gene and of the latter, insertion of a tumor- or tissue-specific promoter [3,4]. Traditionally, recombinant HSV vectors have been generated through homologous recombination by co-transfecting mammalian cells with purified HSV DNA and a plasmid containing exogenous sequences flanked by viral sequences homologous to the insertion site. This method usually requires laborious screening or selection and can take several months. Recent advances in bacterial artificial chromosome (BAC) technology have enabled cloning of the whole HSV genome as a BAC plasmid and subsequent manipulation in *E. coli *[5-8]. Thus, we sought a method to generate recombinant oncolytic HSV vectors more easily and quickly using BAC technology.

It is not practical to manipulate BAC-cloned HSV DNA using conventional subcloning methods such as restriction enzyme digestion and ligation, because HSV-BAC DNA is usually more than 150 kilobases (kb) in length. To circumvent this problem and achieve efficient and accurate manipulation in *E. coli*, site-specific recombination (e.g. Cre/loxP system), homologous recombination/allelic exchange (e.g. RecA, RecE/T, bacteriophage λ *red *system), and transposon integration have been used for mutagenesis or the introduction of foreign sequences into BAC-cloned virus genomes [6,8-16]. Another factor to consider in BAC-mediated recombinant HSV construction is the packaging capacity of the HSV virion. It has been reported that packaging of DNA fragments larger than 156 kb into HSV virions is not efficient [17,18]. BAC cloning requires insertion of mini F plasmid sequences and antibiotic resistance genes into the viral genome and the length of these BAC backbone sequences is usually greater than 6 kb in total [19]. Insertion of BAC sequences into the wild-type HSV genome (152 kb) will increase the genome length to ~158 kb, and there will be no space left for the insertion of additional sequences. To avoid deleterious effects of the BAC sequences, including growth defects and potential transmission between bacteria and man, some herpesvirus BAC clones have been constructed with loxP site-flanked BAC sequences, which can be removed by Cre recombinase [8,20,21]. In this study, we chose to use two independent site-specific recombination systems, Cre/loxP and FLP/FRT, sequentially in two separate steps. These two steps are: (*i*) integration of the shuttle vector containing a foreign sequence into the HSV-BAC genome and (*ii*) excision of the BAC sequences from the integrated HSV-BAC-shuttle DNA. We also employed another strategy for efficient generation of correctly recombined HSV vectors. The size of the integrated HSV-BAC-shuttle DNA was designed to exceed the packaging capacity of the HSV virion by inserting a stuffer sequence, so that recombinant HSV genomes are efficiently packaged and propagated only after removal of the BAC and stuffer sequences by FLPe. This strategy enabled us to obtain nearly pure (>99%) preparations of correctly recombined virus lacking the BAC sequences by simply co-transfecting mammalian cells with the integrated HSV-BAC-shuttle vector and an FLPe expression plasmid. We termed this system the Flip-Flop (FLP/FRT and Cre/loxP mediated) HSV-BAC system.

In this paper, we describe the Flip-Flop HSV-BAC system and its use for the generation of recombinant HSV vectors with the essential ICP4 gene driven by the CMVIE promoter. ICP4 (infected cell protein 4) is the product of the immediate-early α4 or IE-3 gene, and functions as the main transcriptional activator of viral genes [22,23]. Previously, we reported the generation of transcriptionally-targeted oncolytic HSV vectors using an ICP4-deletion mutant HSV d120 as a backbone [4]. We introduced an albumin-promoter-driven ICP4 expression cassette into the *tk *gene locus of d120 and showed that the resultant virus G92A selectively replicated in and killed hepatoma cells and tumor xenografts in which the albumin promoter was active [24]. Using the same strategy, another group generated an oncolytic HSV vector targeting soft tissue and bone tumors with an ICP4 cassette driven by human calponin promoter [25]. For the present study, we cloned d120 as a BAC plasmid, with the BAC sequences integrated in the UL39 (ICP6) gene, and then inserted ICP4 expression cassettes into d120-BAC using the Flip-Flop HSV-BAC system. ICP6 is the large subunit of viral ribonucleotide reductase and disruption of this gene limits HSV growth to actively dividing cells [26-28]. We showed that insertion of an ICP4 expression cassette driven by the CMVIE promoter efficiently drove viral replication, whereas insertion of the ICP4 coding sequence (CDS) alone did not restore viral replication. Use of a tumor-specific promoter instead of the CMVIE promoter, should generate a transcriptionally-targeted oncolytic HSV vector. Furthermore, these studies illustrate the power of the Flip-Flop HSV-BAC system to rapidly generate HSV recombinants.

## Results

### Outline of the Flip-Flop HSV-BAC system

**Figure 1:**
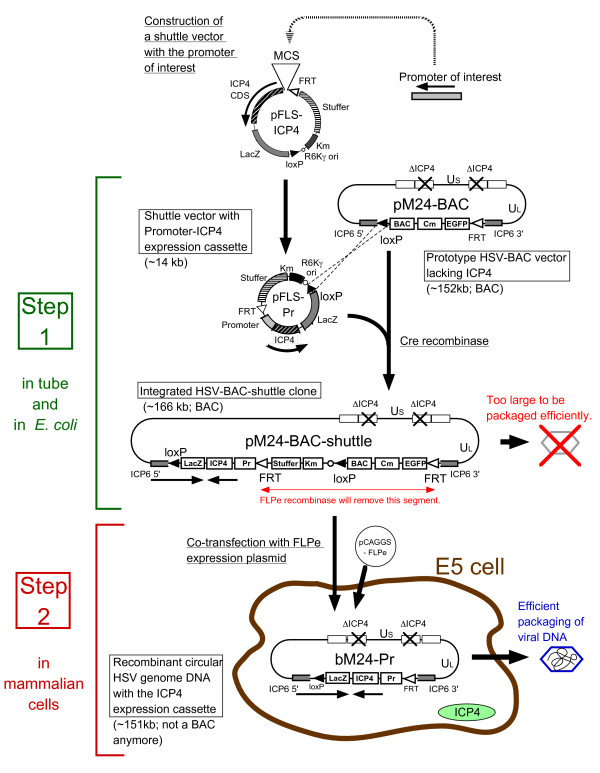
**Schematic outline of the Flip-Flop HSV-BAC system for the generation of exogenous promoter driven HSV vectors**. Prior to recombinant virus construction, a promoter of interest (Pr) is inserted into the MCS of the promoterless shuttle vector pFLS-ICP4 to generate the ICP4 expression cassette driven by that promoter. The prototype HSV-BAC vector plasmid, pM24-BAC contains the genome of replication-deficient ICP4-deletion mutant d120. In the first step of the Flip-Flop HSV-BAC system, the shuttle vector pFLS-Pr is integrated into the loxP site on pM24-BAC, using Cre recombinase, and then electroporated into *E. coli *to obtain the integrated pM24-BAC-shuttle. The second step is performed in mammalian E5 cells, which carry the ICP4 gene and are permissive for ICP4- mutant replication. When FLPe recombinase expression plasmid pCAGGS-FLPe is co-transfected with pM24-BAC-shuttle to E5 cells, the FRT-flanked segment on the oversized vector (marked with orange arrow), containing the BAC vector and stuffer sequences, is excised by the recombinase. The reduction in genome size permits efficient packaging and production of the recombinant virus (bM24-Pr). As pM24-BAC-shuttle is oversized (>166 kb), the vector containing BAC sequences cannot be packaged efficiently into the HSV virion. BAC: BAC backbone and replication origin (mini F plasmid sequences), Cm: Chloramphenicol resistance gene, EGFP: Enhanced Green Fluorescence Protein gene, Km: Kanamycin resistance gene, LacZ: β-galactosidase gene, Stuffer: Stuffer sequence from bacteriophage lambda, ICP4: HSV ICP4 coding sequence, Pr: Exogenous promoter of interest, US: Unique short sequence of HSV, UL: Unique long sequence of HSV, Open circle: R6Kγ plasmid replication origin, ICP6 5': 5' portion of the HSV ICP6 coding sequence, ICP6 3': 3' portion of the HSV ICP6 coding sequence, Closed triangle: loxP recombination site, Open triangle: FRT recombination site.

**Figure 2:**
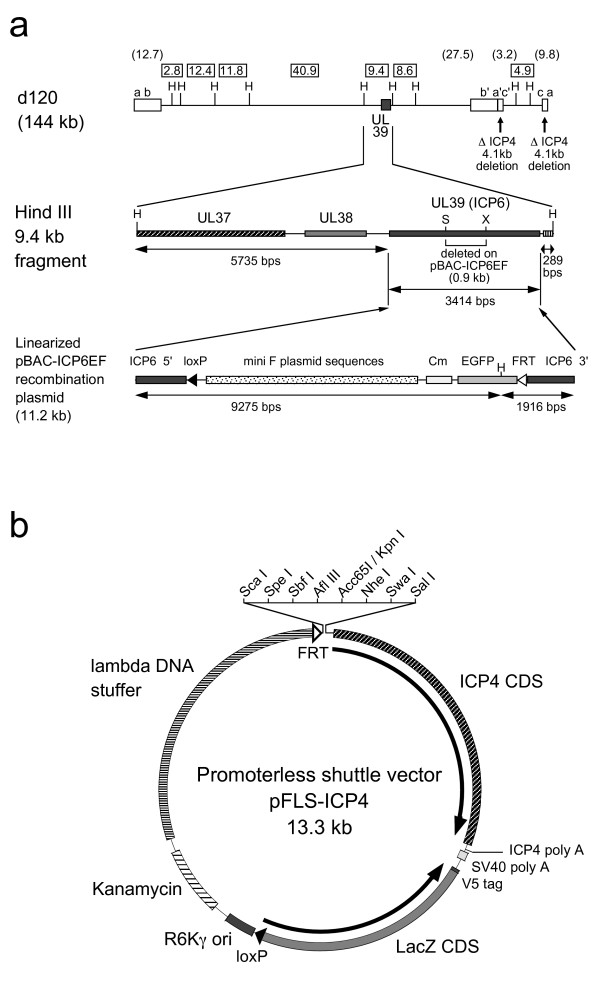
**Schematic representation of the prototype recombinant virus M24-BAC and the promoterless shuttle vector pFLS-ICP4**. (**a**) M24-BAC recombinant virus has the BAC sequences inserted into the UL39 (ICP6) gene of mutant HSV d120. (upper) d120 has a 4.1 kb deletion in both copies of the diploid ICP4 gene. (middle) The ICP6 gene coding the large subunit of viral ribonucleotide reductase is located in the 9.4 kb HindIII restriction fragment of the HSV genome. (lower) The BAC vector sequences were inserted into the d120 genome between the StuI and the XhoI sites of UL39 via homologous recombination using the recombination plasmid pBAC-ICP6EF. The mini F plasmid sequences contain four regulatory genes (*oriS*, *repE*, *parA*, and *parB*) essential for replication and copy number control of the plasmid [19]. H, HindIII; S, StuI; X, XhoI; Open boxes, HSV inverted repeats. The numbers in rectangles at the top of the figure show the lengths in kb of HindIII restriction fragments of d120. The numbers in parentheses show the lengths in kb of terminal restriction fragments. (**b**) Schematic map of the promoterless shuttle vector pFLS-ICP4. MCS is situated upstream of the HSV ICP4 coding sequence so that an exogenous promoter can be inserted to drive ICP4 expression.

The Flip-Flop HSV-BAC system for the generation of exogenous promoter driven HSV vectors is illustrated in Figure 1. The two starting plasmids for this system are the prototype HSV-BAC clone, pM24-BAC, and the promoterless shuttle vector pFLS-ICP4, both of which carry one loxP and one FRT recombination site. pM24-BAC contains the genome of d120 virus with the HSV ICP6 gene (UL39) disrupted by insertion of the BAC cassette (Fig 2a). The shuttle plasmid pFLS-ICP4 contains the ICP4 CDS with a multiple cloning site (MCS) upstream so exogenous promoters can be inserted to drive ICP4 expression, the R6Kγ origin of replication, kanamycin resistance gene, LacZ marker gene, and a stuffer sequence derived from bacteriophage lambda (Fig 2b). Promoters of interest (Pr) are inserted into the MCS by conventional subcloning methods to create a shuttle plasmid containing an ICP4 expression cassette driven by that promoter (pFLS-Pr) (Fig 1).

In the first step, the shuttle vector pFLS-Pr is integrated into pM24-BAC by Cre recombinase *in vitro *and an integrated BAC clone (pM24-BAC-shuttle) isolated in *E. coli *by selection with kanamycin and chloramphenicol. As replication from the R6Kγ origin is dependent on the π protein encoded by the *pir *gene and the BAC host *E. coli *strain is *pir*-, the R6Kγ origin in the integrated BAC does not interfere with BAC replication [29]. In the second step, the integrated pM24-BAC-shuttle DNA and the FLPe expression plasmid are co-transfected to ICP4-complementing E5 cells and the BAC backbone and stuffer sequences, flanked by FRT sites derived from pM24-BAC and the shuttle vector, are excised by FLPe recombinase (Fig 1). Any HSV genome retaining the BAC sequences (from the original pM24-BAC-shuttle DNA) is oversized and cannot be packaged efficiently.

### Cloning of the prototype HSV-BAC clone pM24-BAC

**Figure 3:**
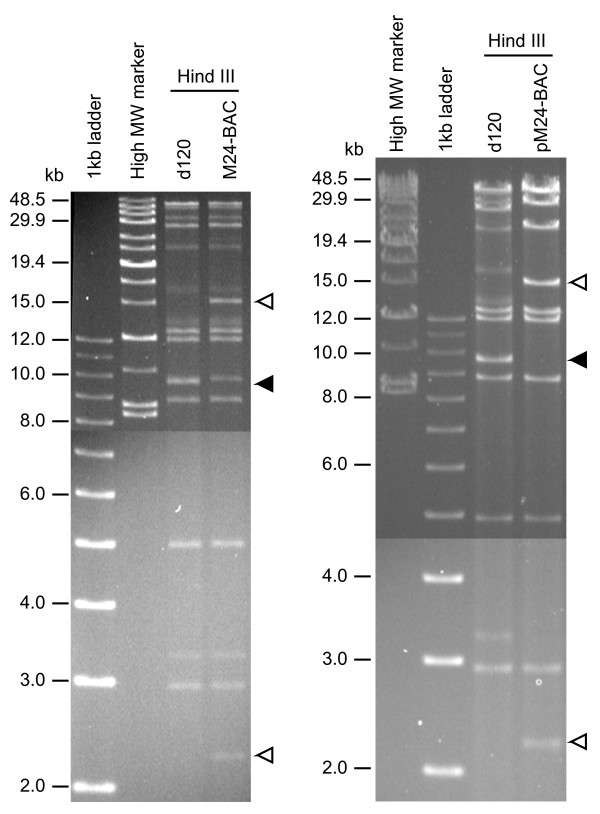
**Genomic structure of M24-BAC virus and pM24-BAC plasmid DNA**. HindIII restriction analysis of M24-BAC virus DNA (left panel) and pM24-BAC plasmid DNA (right panel) is shown. Closed arrowhead: 9.4 kb HindIII fragment of d120 (containing ICP6 region), Open arrowheads: 15 kb and 2.2 kb HindIII fragments generated by insertion of the BAC sequences. A band present in M24-BAC digest just above the closed triangle is the submolar terminal restriction fragment (9.8 kb) derived from the short terminal repeat. This band is overlapped by the 9.4 kb restriction fragment of d120 and absent from pM24-BAC. The restriction pattern of pM24-BAC indicates that this clone contains both the UL and US sequences of HSV genome in the forward orientation. Other bands present in the M24-BAC digest but not in pM24-BAC are submolar terminal fragments or L-S junction fragments derived from other isomeric forms of virus genome.

To initiate HSV-BAC cloning, a recombinant HSV mutant containing BAC sequences was generated by homologous recombination. The BAC cassette was inserted into the ICP6 (UL39) locus of ICP4-deletion mutant d120 (Fig 2a). Resultant ICP4/ICP6 double-deletion mutant HSV (M24-BAC) formed EGFP-positive plaques, somewhat smaller than those of parental d120, presumably due to the additional ICP6 deletion (data not shown). The genomic structure of M24-BAC was confirmed by HindIII restriction analysis (Fig 3; left panel).

An HSV-BAC plasmid containing the whole genome of M24-BAC, pM24-BAC, was isolated by transforming DH10B *E. coli *with circular viral DNA and its genomic structure confirmed by HindIII restriction analysis (Fig 3; right panel). pM24-BAC DNA was infectious, generating virus, bM24-BAC (b refers to BAC/bacterial derived virus) after transfection of E5 cells. Two independent plaque-purified bM24-BAC isolates and parental M24-BAC had similar properties: (*i*) they formed comparably sized EGFP-positive plaques; (*ii*) they had identically sized restriction endonuclease fragments [see Additional file 1]; and (*iii*) they had identical virus growth kinetics and similar virus yields [see Additional file 2]. Therefore, we concluded that the reconstituted bM24-BAC virus retains the same characteristics as the original M24-BAC virus and that the HSV-BAC plasmid pM24-BAC contains a faithful copy of the M24-BAC virus genome.

### Shuttle vector integration into pM24-BAC by Cre recombinase

**Figure 4:**
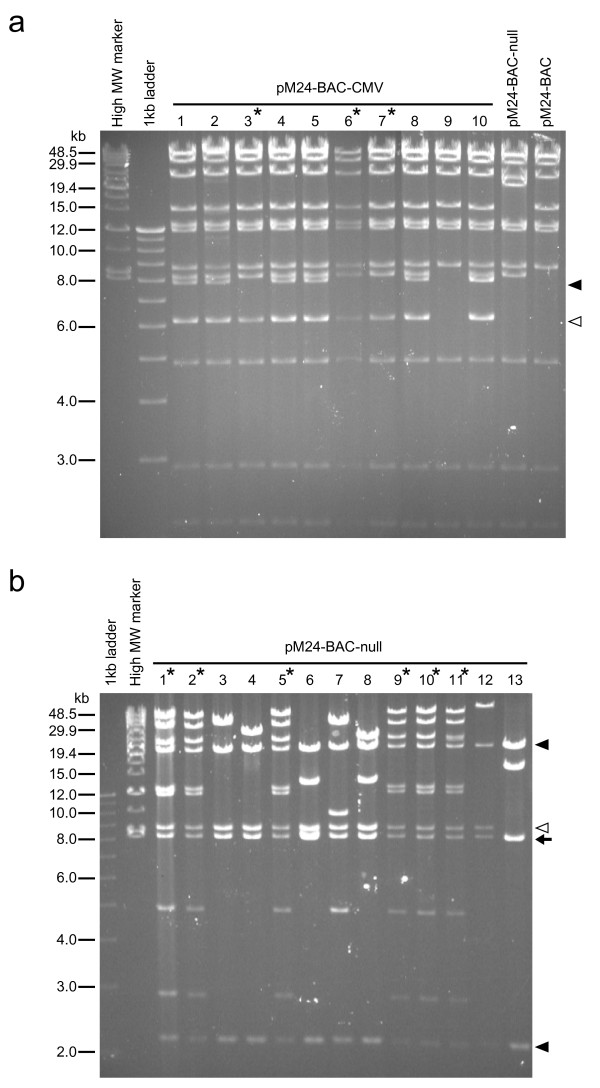
**Analysis of BAC clones after site-specific recombination**. (a) HindIII restriction analysis of chloramphenicol/kanamycin double-resistant clones (pM24-BAC-CMV) after Cre-mediated integration between pM24-BAC and pFLS-CMV shuttle vector. Clones #3, 6, and 7 (marked with asterisks) show the expected restriction pattern. Clones #1, 2, 4, 5, and 8 contain an additional 7.8 kb fragment (*closed arrowhead*) and a greater amount of the 6.2 kb fragment (*open arrowhead*) which is consistent with the expected digestion pattern of pM24-BAC-CMV with doubly inserted shuttle vector [see Additional file 3]. Clone #10 also contains a greater amount of the 7.8 kb fragment, suggesting insertion of three or more copies of the shuttle vector. The remaining clone (#9) did not undergo recombination and shows a pattern identical to that of pM24-BAC. (b) HindIII restriction analysis of pM24-BAC-null clones obtained after Cre-mediated integration between pM24-BAC and pFLS-XICP4 shuttle vector. Clones #1, 2, 5, 9, 10 and 11 (marked with asterisks) show a restriction pattern consistent with singly integrated pM24-BAC-null. The other seven clones contain a partially deleted, incomplete HSV-BAC genome. The 8.2 kb HindIII fragment containing the BAC backbone (*arrow*) and the two neighboring fragments (20.1 and 2.2 kb; *closed arrowheads*) are preserved in all clones, and the 8.6 kb fragment (*open arrowhead*) adjacent to the 2.2 kb fragment is preserved in all clones except clone #13. Other fragments are lost in the deletion clones and new fragments of varying lengths are observed, suggesting that these deletions occurred randomly at different locations. For the HindIII restriction map, see Fig 2a and Additional file 3.

As a proof-of-principle and to validate the Flip-Flop HSV-BAC system, we constructed three recombinant viruses (Table 1): (*i*) bM24-CMV, with the ICP4 CDS driven by the human CMVIE promoter, (*ii*) bM24-null, with the ICP4 CDS lacking a promoter, and (*iii*) bM24-empty, with no ICP4 CDS, which was generated with shuttle vector pFLS2 lacking the ICP4 CDS. First, the specificity and efficiency of Cre recombinase mediated integration into pM24-BAC (Step1) was examined. In the pM24-BAC-CMV cloning, three BAC clones (clones #3, 6, 7; Fig 4a, marked with asterisks) contained a single integrated copy of pFLS-CMV, whereas six other clones (#1, 2, 4, 5, 8, 10) had an additional 7.8 kb band (Fig 4a* closed arrowhead*), indicating integration of multiple copies of the shuttle vector (for HindIII restriction map, see Fig 2a and Additional file 3). The remaining one clone (#9) had not undergone recombination and showed a pattern identical to that of pM24-BAC. We used the single insert clones for bM24-CMV virus generation. In the pM24-BAC-null cloning, six out of 13 BAC clones contained the complete HSV-BAC genome based on HindIII restriction analysis, all of which carried a single integrated copy of the shuttle vector, whereas the remaining seven clones contained a partially deleted, incomplete HSV-BAC genome (Fig 4b). These deletions seemed to have occurred randomly at different locations, presumably due to shearing during DNA manipulation (Step1). In the pM24-BAC-empty cloning, nine out of ten clones contained the complete HSV-BAC genome, all of which carried multiply integrated pFLS2 shuttle vector (data not shown). Even with multiple copies of the shuttle vector integrated into the loxP site of pM24-BAC, FLPe recombination between the most distal pair of FRT sites during virus generation should remove any extra copies of the shuttle vector along with the BAC backbone sequences [see Additional file 3]. Thus, these multiple insert clones were used to create bM24-empty virus.

### Recombinant virus generation by FLPe recombinase excision of BAC sequences

**Figure 5:**
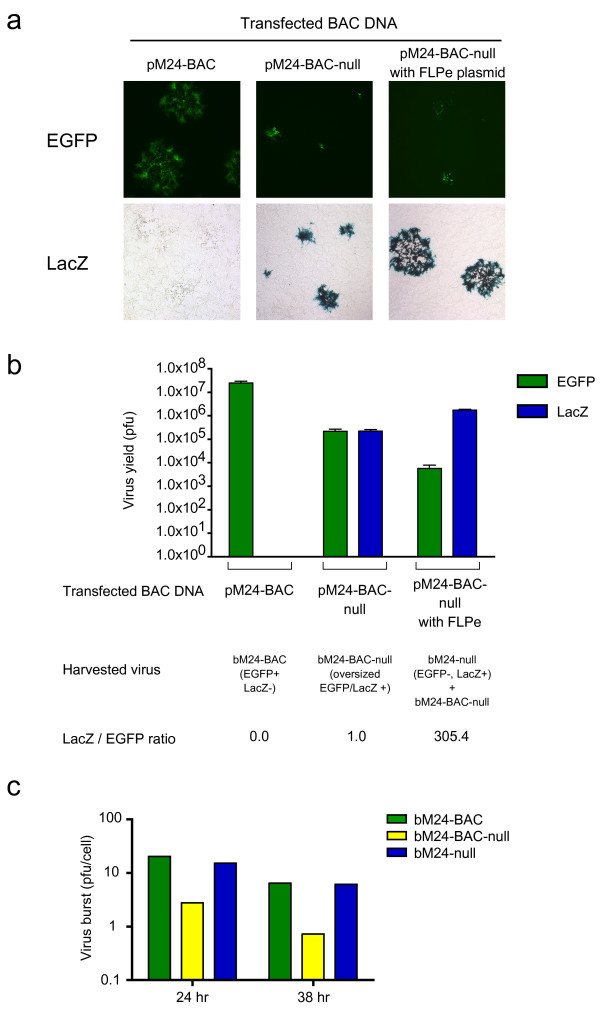
**Recombinant virus generation after FLPe recombinase excision of BAC sequence**. (a) EGFP and LacZ marker expression by recombinant viruses harvested after transfection of pM24-BAC (prototype HSV-BAC) or pM24-BAC-null DNA with or without the FLPe recombinase plasmid. E5 cells were transfected with indicated DNA and 6 days after transfection, viruses were harvested and titered on E5 cells. Detection of EGFP was performed without fixation before LacZ staining. When pM24-BAC alone was transfected, all harvested viruses formed EGFP-positive plaques none of which were LacZ positive. When pM24-BAC-null alone was transfected, the viruses formed both EGFP- and LacZ-positive plaques. Sizes of the plaques were smaller than those of viruses harvested from pM24-BAC transfected cells. When pM24-BAC-null and pCAGGS-FLPe were co-transfected, all harvested viruses formed LacZ-positive plaques and most of them were EGFP-negative. A very few number of EGFP-positive plaques, which were smaller in size than EGFP-negative plaques, were observed in lower dilution wells. All photos are at same magnification. (b) Titer of EGFP-positive and LacZ-positive viruses harvested in each transfection group (error bars show standard deviation. N = 3). Ratios between titers of EGFP-positive and LacZ-positive virus in each group are shown below. (c) Single-step growth assay of viruses harvested in Fig 5b. 8 × 10^3 ^of E5 cells plated in 48-well plates were infected at an MOI of 2.5. Progeny viruses were harvested 24 or 38 hours after infection and titered on E5 cells. The virus yield was divided by the number of infected cells (Virus burst).

**Figure 6:**
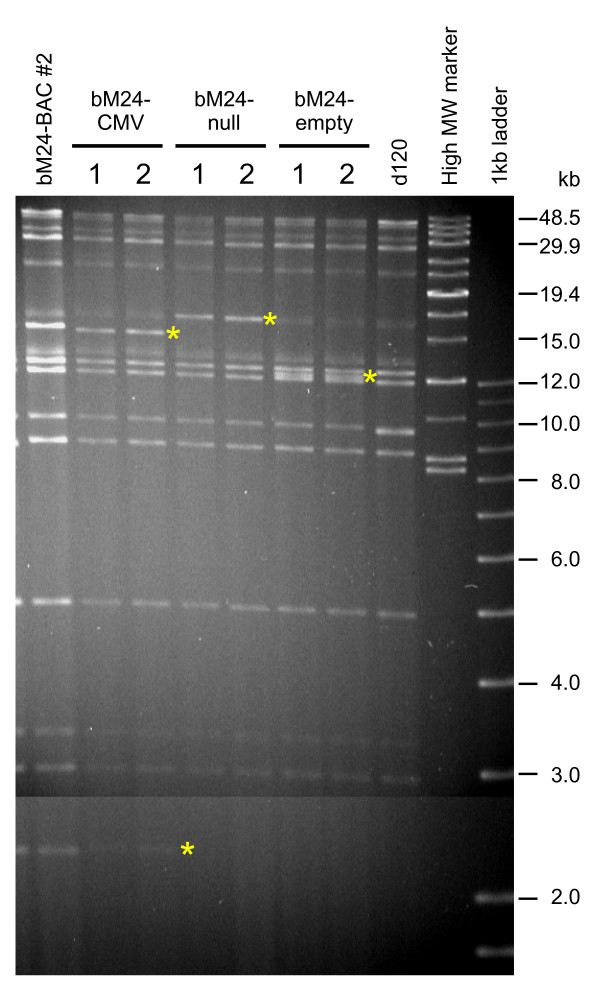
**Analysis of genomic DNA from recombinant viruses generated using the Flip-Flop HSV-BAC system**. DNA from two independent isolates was purified, digested with HindIII and electrophoresed on an agarose gel. The DNAs of parental viruses bM24-BAC and d120 illustrate the common fragments, while the asterisks denote fragments containing inserts derived from the shuttle vector. [see Additional file 3].

Next, we compared the efficiency of recombinant virus generation following transfection of pM24-BAC-shuttle DNA with or without the FLPe plasmid to determine whether the increased size was sufficient to inhibit packaging of BAC-containing viral genomes. E5 cells were transfected with: (*i*) the prototype HSV-BAC, pM24-BAC (152 kb) alone, (*ii*) pM24-BAC-null alone (166 kb or approximately 14 kb larger than the wild-type HSV genome), or (*iii*) pM24-BAC-null and the FLPe expression vector (pCAGGS-FLPe). Progeny viruses were harvested 5 days after transfection and titered on E5 cells. As illustrated in Figure 1, expression of LacZ indicates the precise integration of the shuttle vector into pM24-BAC and the lack of EGFP expression, the loss of the BAC vector sequences. When pM24-BAC alone was transfected, the virus yield was 2.5 × 10^7 ^pfu and all harvested viruses formed EGFP-positive and LacZ-negative plaques (Fig 5a, b). On the other hand, the titer after pM24-BAC-null transfection (bM24-BAC-null virus) was two logs lower (2.2 × 10^5 ^pfu). Furthermore, the virus formed much smaller plaques than pM24-BAC derived virus and almost all of the plaques were both EGFP- and LacZ-positive, as would be expected. When pM24-BAC-null was co-transfected with the FLPe expression plasmid, most of the harvested viruses formed EGFP-negative/LacZ-positive plaques and their sizes were comparable to those of the viruses derived from pM24-BAC (Fig 5a, b). There was a very low proportion of EGFP/LacZ double-positive plaques (5.7 × 10^3 ^pfu, compared to 1.7 × 10^6 ^pfu for single LacZ-positive), similar in size those formed by bM24-BAC-null. Thus, more than 99% of the harvested viruses were EGFP-negative/LacZ-positive. Similar results were obtained in the generation of bM24-CMV and bM24-empty viruses. In order to confirm the impaired growth of the oversized virus carrying the BAC and stuffer sequences, we performed single-step growth assay using the crude virus preparations harvested in Fig 5b. While the virus burst sizes of bM24-BAC and bM24-null were similar, that of bM24-BAC-null was 5-7-fold less (Fig 5c). Two independently generated isolates (designated as #1 and #2) of the EGFP-negative/LacZ-positive virus were plaque-purified and their genomic structure confirmed by restriction endonuclease analysis (Fig 6).

### The recombinant viruses replicate efficiently only when ICP4 is expressed

**Figure 7:**
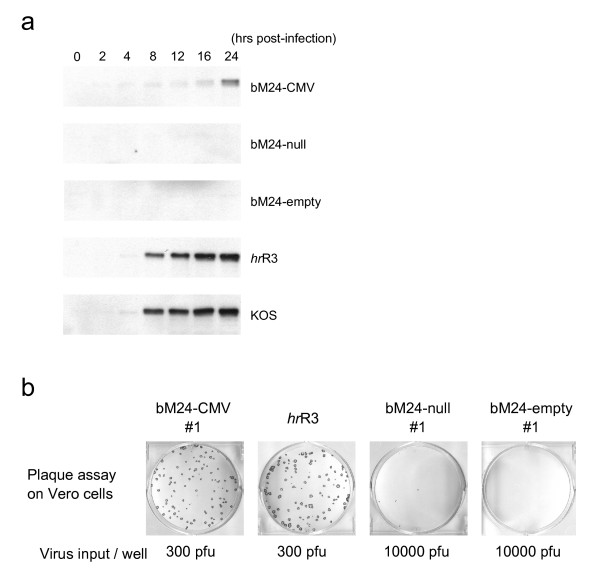
**Phenotype of recombinant viruses generated by the Flip-Flop HSV-BAC system**. (**a**) Time course of ICP4 expression in Vero cells infected with recombinant viruses. bM24-CMV induced weak expression of ICP4 as early as 4 hours post-infection (pi), which increased over time up to 24 hours pi. Vero cells infected with bM24-null and bM24-empty showed no detectable ICP4 expression. *hr*R3 and KOS which retain two copies of the wild-type ICP4 gene showed very strong expression of ICP4 after 8 hours pi. (**b**) Plaque forming assay on Vero cells. Vero cells grown in 6-well dishes were infected with the indicated amounts of recombinant virus, fixed five days after infection and stained with X-gal. A few tiny plaques are visible in the bM24-null well.

**Figure 8:**
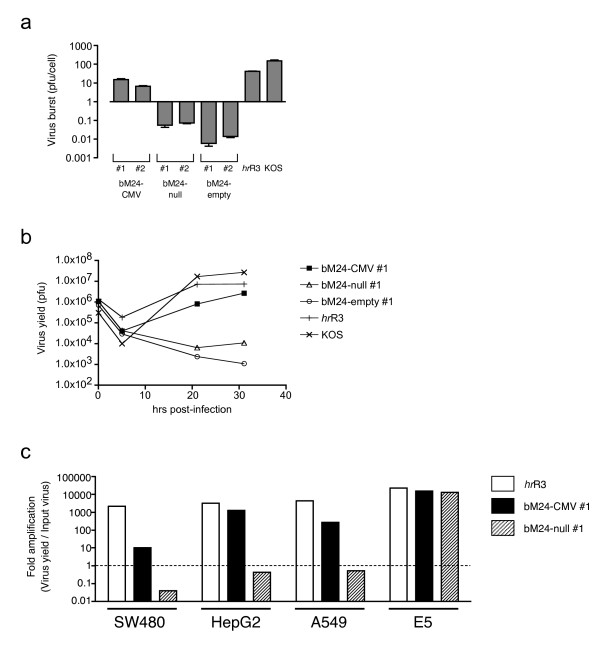
**Replication of recombinant viruses in Vero and human tumor cells**. (**a**) The virus burst on Vero cells. 1.8 × 10^5 ^cells were infected with the indicated recombinant viruses (2 independent isolates each) at an MOI of 3 and viruses harvested 31 hours pi and titered. The virus yield was divided by the number of infected cells. Error bars show standard deviation (N = 3). (**b**) Time course of virus replication on Vero cells, from a similar experiment as in a, except virus was harvested at the indicated times post-infection. (**c**) Replication assay of bM24-CMV, bM24-null and *hr*R3 viruses on different tumor cell lines. Cells were infected with 10^4^ pfu of the indicated viruses (MOI~0.01) and virus harvested 72 hours pi. Fold amplification of virus (Virus yield/input virus) is shown, with 1 being the amount of input virus. bM24-CMV grew in all tumor cell lines in varying degree, whereas bM24-null grew only on ICP4-complementing E5 cells. *hr*R3 grew to a similar degree in all three tumor cell lines.

In order for pM24-BAC to be a suitable backbone vector for the generation of transcriptionally-targeted HSV vectors, viruses generated from it should not replicate in non-complementing cells in the absence of an active upstream promoter. This was tested in Vero cells, which are the parental cell line for the ICP4-complementing E5 cells, by comparing the growth of bM24-CMV to bM24-null and bM24-empty. The expression of ICP4 was detected in cells infected with bM24-CMV as early as 4 hours post-infection and increased up to 24 hours at least, but not in cells infected with bM24-null or bM24-empty (Fig 7a). This is similar kinetics to that seen after KOS (wild-type HSV strain) or *hr*R3 (ICP6- mutant strain) infection, but at much lower levels (Fig 7a). bM24-CMV formed plaques efficiently on Vero cells, similar to *hr*R3, while only a few small plaques were formed after infection with over 30-fold more bM24-null and no plaques with bM24-empty (Fig 7b). In a virus burst assay, bM24-CMV yielded ~5–10 pfu/cell, while bM24-null and bM24-empty yielded 0.1 and 0.01 pfu/cell, respectively (Fig 8a). The replication of bM24-CMV was delayed compared to *hr*R3 or KOS (Fig 8b), probably reflecting the limited expression of ICP4. However, this level of ICP4 expression is sufficient to drive viral replication. Interestingly, there was over a 100-fold difference in bM24-CMV virus yield in different human tumor cell lines, with HepG2 the most susceptible and SW480 the least, whereas *hr*R3 replication was similar in all 3 tumor cell lines and bM24-null did not appreciably replicate in any of the cells (Fig 8c).

## Discussion

Transcriptionally-targeted oncolytic virus vectors are promising cancer therapeutic agents and some of them have already been applied to cancer patients in clinical trials. For example, an oncolytic adenovirus vector CV706 whose replication is driven by the prostate-specific antigen (PSA) promoter/enhancer element has completed Phase I clinical trials and some of the patients showed a significant decrease in serum PSA levels [30]. Although there are many potentially promising tumor-specific promoters reported in the literature and many of them have been used for generation of oncolytic adenoviruses [31], only a few have been tested in the context of transcriptionally-targeted HSV vectors due to the extensive labor involved in constructing recombinant HSV vectors [4,25,32].

Traditionally, recombinant HSV vectors have been generated via homologous recombination between purified HSV DNA and recombination plasmid in co-transfected cells. In addition to the inefficiency of recombination, there is the need to screen or select plaques for the correct recombinant. This has hampered the development of new recombinant HSV vectors. Recent advances in BAC technology have enabled the cloning of herpesvirus genomes as BACs and their manipulation in *E. coli *[5-14,16,20,21]. Using our Flip-Flop HSV-BAC system, once a shuttle vector containing a promoter of interest is constructed, nearly pure (>99%) preparations of recombinant vector can be obtained typically within two weeks, which can be easily plaque-purified subsequently. It should be noted that this system can also be applied to the generation of transgene-expressing HSV vectors if replication-competent HSV-BAC is used as a prototype BAC [33]. A similar strategy for the generation of oncolytic HSV vectors, termed the HSVQuik system, has recently been reported [34].

In Step1 of the Flip-Flop HSV-BAC system, Cre-mediated recombination was performed biochemically and then integrated BAC DNA electroporated into *E. coli*. As high-molecular-weight BAC DNA is vulnerable to mechanical shearing, these procedures could cause double-strand breaks, lowering the proportion of correctly integrated HSV-BACs. When we generated pM24-BAC-null clones, more than half of the clones (7 out of 13) had suffered large deletions occurring at random locations (Fig 4b), while for pM24-BAC-CMV and pM24-BAC-null cloning, 90% (9 out of 10) of the BAC clones contained a full-length HSV-BAC genome, with either single or multiple shuttle inserts (Fig 4a and data not shown). To date, we have generated several different transcriptionally-targeted HSV vectors using this system and found that the efficiency of full-length HSV-BAC cloning after Cre recombination varied between 40 to 100% (average 69%; 67 complete clones out of 97 clones) (TK, unpublished data). An alternate way to avoid BAC DNA shearing in Step1 would be to perform site-specific recombination in bacteria by introducing the shuttle and Cre-expressing plasmids into the *E. coli *carrying the prototype HSV-BAC plasmid, as reported previously [35]. Terada et al. employed, in their HSVQuik system, a similar strategy using an FLP-expressing plasmid for the integration of their HSV-BAC and transfer plasmids, and reported that ~80% of HSV-BAC clones contained correct co-integrants [34].

The efficiency of FLPe-mediated excision of the BAC and stuffer sequences from the integrated HSV-BAC-shuttle DNA was high, with over 99% of recombinant virus harvested after Step2 LacZ-positive and EGFP-negative. In order for recombined virus to be generated, several events need to occur: (*i*) both the integrated HSV-BAC-shuttle and the FLPe plasmid is transfected into a same cell, (*ii*) FLPe is expressed, (*iii*) the BAC sequences are excised from the HSV-BAC DNA, (*iv*) the recombined viral genome is replicated and (*v*) packaged. There are multiple factors affecting these processes, including the rate and level of FLPe expression and the timing of recombination. In fact, we do not know at which stage the FLPe recombination occurs, i.e. in the original HSV-BAC DNA or in the replicated virus DNA concatemer. In the former case, all the replicated viral DNA are correct recombinants and readily packaged, whereas in the latter case, only a fraction of the viral DNA concatemer may be packaged. Therefore, the efficiency of viral production through all these events could be lower than that after transfection of pM24-BAC alone, from which bM24-BAC virus can be generated directly without recombination. In our experiment, the yield of FLPe-recombined virus was sufficient, albeit a log lower than that of bM24-BAC (Fig 5b). For the generation of bM24-empty virus, we used pM24-BAC-empty clones that contained multiple integrated copies of shuttle vector. Even in these multiple inserts, FLPe recombination between the most distal pair of FRT sites results in the generation of correct recombinant virus, while recombination between other pairs of FRT sites generates viral genomes, which are still oversized and cannot be efficiently packaged. Thus, multiple-insert BAC clones can be used for recombinant virus generation.

When pM24-BAC-null was co-transfected with the FLPe plasmid to excise the BAC sequences, the ratio of bM24-null to bM24-BAC-null was over 300 (Fig 5b). There are two factors affecting the final yield ratio between the recombined and non-recombined virus: (*i*) efficiency of recombination, and (*ii*) difference in growth kinetics between the two viruses. Previously, Smith and Enquist, reported that co-transfection of loxP-carrying PRV-BAC DNA and a Cre expression plasmid resulted in a virus preparation containing 10–15% non-recombined viruses [21]. In order to improve the yield of correctly recombined virus, they inserted a Cre expression cassette into the loxP-flanked BAC cassette so that Cre was expressed from non-recombined BAC-virus. This self-excision strategy worked very well and the yield of correctly recombined virus was more than 99.9%. In the Flip-Flop HSV-BAC system, we took a different approach to maximize the yield of correct recombinant virus; a stuffer sequence was included in the shuttle vector so that the integrated HSV-BAC clones are oversized and produce virus efficiently only after the BAC and stuffer sequences are removed. Using this strategy, ~99.7% of harvested viruses were correct recombinants (Fig 5b). As shown in Fig 5c, the growth of oversized bM24-BAC-null virus (~166 kb) was much reduced compared to that of recombined virus bM24-null. As HSV-BAC can accommodate larger DNA, an integrated HSV-BAC with a much larger genome size (e.g. >180 kb) using a longer stuffer sequence, should further reduce the production of non-recombined virus.

We previously reported the generation of albumin-promoter-driven HSV vector G92A in which the ICP4 expression cassette was inserted into the thymidine kinase (*tk*) gene [4]. Unfortunately, *tk*-mutant viruses are resistant to acyclovir treatment, which is not desirable for clinical application [36]. To avoid this problem, we chose to insert the BAC vector sequences and ICP4 expression cassette into the UL39 gene, preserving the *tk *gene. UL39 encodes ICP6, the large subunit of the viral ribonucleotide reductase, and its inactivation limits vector replication to actively dividing cells [26-28]. Additionally, ICP6 inactivation increases virus sensitivity to acyclovir [37,38]. It is worth noting that the initial HSV-BAC construct can be generated by insertion of the BAC cassette into any desired sequence or gene in the viral genome, other than ICP6.

In the current study, it was important to first demonstrate that there were not cryptic regulatory sequences that would drive ICP4 expression in the absence of promoters when inserted into the ICP6 region of HSV. bM24-null is such a construct and was highly defective in replication, only minimally better than bM24-empty, lacking any ICP4 sequences. These results show that pM24-BAC is suitable as a prototype construct for the generation of transcriptionally-targeted HSV vectors. In a plaquing assay, bM24-null formed a few plaques on Vero cells when infected at 10000 pfu/well, while no plaques were formed by the same amount of bM24-empty (Fig 7b). These plaques might have arisen from bM24-null mutants with genome rearrangements or deletions that activated ICP4 expression.

When ICP4 was expressed under the control of the CMVIE promoter, its level of expression was much lower than KOS and *hr*R3, which have diploid copies of wild-type ICP4 gene (Fig 7a). Recently, Terada et al. reported that HSV promoters, ICP6, IE2 and IE4/5, induced a higher level of luciferase expression compared to the CMVIE promoter when inserted in the ICP6 gene [34], the same location as in bM24-CMV. Regulation of HSV transcription and translation is complex, with ICP4 playing a major role, both as a trans-activator and repressor [23]. Upon infection, the tegument protein VP16 activates HSV immediate-early gene expression, however it does not similarly activate the CMVIE promoter [39], which could account for the delayed kinetics. In a study of CMVIE promoter-GFP expression in HSV immediate-early gene mutants, it was found that CMVIE promoter activity was dependent upon ICP0 expression levels [40] and that the CMVIE promoter in the HSV genome was actively repressed in the absence of viral activators [41].

It is not clear what levels of ICP4 expression are necessary for viral replication. In a single-step growth assay (Fig 8a,b), bM24-CMV grew efficiently in Vero cells, with slower kinetics than *hr*R3 or KOS, presumably reflecting the lower and slower induction of ICP4 by the CMVIE promoter. Furthermore, the levels of bM24-CMV replication, as opposed to *hr*R3, varied considerably in different tumor cell lines. This could be due to variable activity of the CMVIE promoter or different levels of ICP4 required for replication in different cell lines. In fact, we found that the transcriptional activity of the CMVIE promoter in SW480 was 3 to 8 fold lower than in HepG2 or A549, using a transient luciferase reporter assay [42]. While considered constitutive, CMVIE promoter activity has been shown to vary greatly in different cells; in transgenic mice, or with transiently transfected plasmids or adenovirus vectors [43-46].

## Conclusion

The Flip-Flop HSV-BAC system we describe enables rapid generation of HSV vectors carrying transgene inserts. By introducing a tumor-specific-promoter-driven ICP4 cassette into pM24-BAC using this system, one should be able to generate transcriptionally-targeted oncolytic HSV vectors. We believe this system will greatly facilitate the screening of a plethora of clinically useful tumor-specific promoters in the context of oncolytic HSV vectors.

## Methods

### Cells and viruses

Vero (African Green Monkey kidney cells), SW480 (Human colon adenocarcinoma), and HepG2 (Human hepatoblastoma) cells were obtained from American Type Culture Collection (Manassas, VA). A549 (Human lung adenocarcinoma) cells were provided by W. Kallas (Massachusetts General Hospital, Boston, MA). E5 cells are Vero cells stably transfected with HSV ICP4, so they express complementing levels of wild type ICP4 upon HSV-1 infection and were provided by N. DeLuca (University of Pittsburgh School of Medicine, Pittsburgh, PA) [22]. Vero and E5 cells were grown in Dulbecco's modified Eagle's medium (DMEM) supplemented with 10% calf serum and other cell lines in DMEM supplemented with 10% fetal calf serum. Wild-type HSV-1 strain KOS was provided by D. Knipe (Harvard Medical School, Boston, MA), ICP6- recombinant *hr*R3 (parental strain KOS) was provided by S. Weller (University of Connecticut Health Center, Farmington, CT) [47], and ICP4 deletion mutant d120 (parental strain KOS) was provided by N. DeLuca [22]. Virus stocks were generated from low-multiplicity infections.

### Plasmids

pCAGGS-FLPe was provided by Dr. P. Lowenstein (Cedars-Sinai Hospital, Los Angeles, CA) [48]. Other plasmids were constructed as follows. *The recombination plasmid, pBAC-ICP6EF:* AscI-tagged ICP6 CDS was generated via PCR from d120 DNA using the LA-PCR kit (TaKaRa Mirus Bio, Madison, WI) and 5'-TATGGCGCGC CAACCCGCCG CGTCTGTTGA AAT-3' (sense) and 5'-ATAGGCGCGC CTTTGTCGGT CACAGCGCGC AGCTC-3' (antisense) primers. The PCR product was digested with AscI, ligated, and digested again with XhoI and StuI. The resultant 2556 bp fragment containing 3' (1.2 kb) and 5' (1.3 kb) fragments of the ICP6 CDS ligated tail-to-head was subcloned into the XbaI/XhoI sites of pBluescriptII-KS+ vector (Stratagene, La Jolla, CA) to create pBS2ICP6TH. The EGFP CDS excised from pEGFP-N1 (Clontech, Palo Alto, CA) was subcloned into the HindIII/AgeI sites of pVP22/myc-His2 vector (Invitrogen, Carlsbad, CA) to create pCMV-EGFP. pCMV-EGFP2 was created by digestion of pCMV-EGFP with PvuII and subsequent self-ligation. Two oligomers containing the FRT recognition sequence (underlined), CGCGCGAAGT TCCTATACTT TCTAGAGAAT AGGAACTTCC TCGAG (sense) and AATTCTCGAG GAAGTTCCTA TTCTCTAGAA AGTATAGGAA CTTCG (antisense) were synthesized, hybridized and subcloned into the MluI/MfeI sites of pCMV-EGFP2 to create pEGFP-FRT. The FRT recognition site followed by the EGFP expression cassette driven by the CMV promoter with the BGH polyA signal was excised from pEGFP-FRT by XhoI and PciI digestion and subcloned into the XhoI/PciI sites of pBSICP6TH to create pICP6TH-EF. Finally, the NotI/PvuII digestion fragment of pICP6TH-EF containing the EGFP cassette, the FRT site and the 3' and 5' fragments of the ICP6 CDS was subcloned into the NotI/SrfI sites of pBelobac11 (Research Genetics, Huntsville, AL) to create pBAC-ICP6EF. *The promoterless shuttle vector, pFLS-ICP4:* Two oligomers containing the loxP recognition sequence (underlined), AGCTTATAAC TTCGTATAAT GTATGCTATA CGAAGTTATC CATGGCTGCA (sense) and GCCATGGATA ACTTCGTATA GCATACATTA TACGAAGTTA TA (antisense) were synthesized, hybridized and subcloned into the HindIII/PstI sites of pUC18 to create pUC-loxP. Then the FRT sequence oligomers described above were subcloned into the BamHI/EcoRI sites of pUC-loxP to create pUC-LF, which was subsequently digested with HindIII and NdeI, filled-in and self-ligated to create pUC-LF2. pCMV-LacZV was constructed from pcDNA6-E/Uni-*lacZ *(Invitrogen) by AgeI and PmlI digestion followed by self-ligation. The NcoI/SalI fragment of pCMV-LacZV containing the LacZ CDS and V5 epitope tag followed by the SV40 polyA signal was subcloned into the NcoI/SalI sites of pUC-LF2 to create pLLZF2. Two oligomers containing a MCS sequence, GATCAGTACT AGTCCTGCAG GCTTAAGGGT ACCGCTAGCA TTTAAATGTC GACAGGCC (sense) and TCGAGGCCTG TCGACATTTA AATGCTAGCG GTACCCTTAA GCCTGCAGGA CTAGTACT (antisense), were synthesized, hybridized and subcloned into the BamHI/SalI site of pLLZF2 to create pLLZMF2. The 4.1-kb SalI/MseI fragment of pGH108 (provided by G. Hayward, Johns Hopkins School of Medicine, Baltimore, MD) [22] containing the ICP4 CDS was subcloned into the StuI/SalI sites of pLLZMF2 to create pLLZMF2-ICP4. To generate a stuffer sequence, the 4.0-kb AgeI-digest fragment of bacteriophage lambda DNA (nucleotide position 6562 – 10550) was obtained by digesting a HindIII digest of lambda DNA (New England Biolab, Beverly, MA). Subsequently, pUni-lmd was created by subcloning the stuffer sequence into the AgeI/XmaI sites of pUni/V5-HisA vector (Invitrogen) that contains the R6Kγ plasmid replication origin and kanamycin resistance gene. Finally, the 7.6-kb HindIII/XhoI fragment of pLLZMF2-ICP4 was subcloned into the NotI/XhoI sites of pUni-lmd to create pFLS-ICP4. *pFLS-CMV:* The CMV promoter was excised from pVP22/myc-His2 by KpnI and ScaI digestion and subcloned into the KpnI/NruI sites of the pFLS-ICP4 to create pFLS-CMV. *pFLS2:* An oligomer containing the NotI recognition sequence, 5'-AGCTCATAGCGGCCGCTATG-3', was synthesized, hybridized and subcloned into the HindIII site of pLLZMF2 to create pLLZMF2N. The 3.5-kb NotI/XhoI digestion fragment of pLLZMF2N was subcloned into the NotI/XhoI sites of pUni-lmd to create pFLS2.

For mammalian cell transfection, plasmid DNA, including BAC, was purified using a plasmid purification kit (QIAGEN, Valencia, CA) following manufacturer's protocols.

### Virus titration

Monolayer cultures of Vero cells (for KOS and *hr*R3) or E5 cells (for ICP4 mutant viruses) grown in six-well dishes were infected with serial dilutions of virus. After removal of virus inoculum, the cells were incubated in DMEM supplemented with 1% IFCS and 0.1% pooled human immune globulin (BayGam; Bayer Corporation, Elkhart, IN) at 37°C for 3 to 4 days until plaques were visible. The cells were fixed with methanol (for Giemsa staining), or 0.5% glutaraldehyde-2% paraformaldehyde (for X-Gal histochemistry). Plaques were counted and the average number of plaques was determined from 2 wells. To count EGFP-positive plaques, cells were observed under an inverted fluorescence microscope without fixation.

### Generation of M24-BAC virus

d120 viral DNA was purified as previously described [49]. Briefly, confluent monolayers of E5 cells were infected with d120 virus at a multiplicity of infection (MOI) of 2, and 16 hours later, when total cytopathic effect (CPE) was observed, cells were harvested, pelleted by centrifugation, washed with Dulbecco's phosphate-buffered saline (PBS), resuspended in RS buffer (10 mM TrisHCl pH8, 10 mM KCl, 1.5 mM MgCl_2_), incubated on ice for 20 min, and homogenized with a Dounce homogenizer. After centrifugation at 1000 *g*, 4°C, 5 min, the pellet containing the nuclei was further lysed in RS buffer with 0.25% TritonX, centrifuged again and the supernatant combined with the initial supernatant, which was treated with Proteinase K (Invitrogen) and SDS at 37°C overnight. Viral DNA was purified by ultracentrifugation in a NaI density gradient [49].

E5 cells (1.8 × 10^5 ^cells/well) plated in six-well dishes were co-transfected with 250 ng of purified d120 DNA and 180 ng of AscI-digested pBAC-ICP6EF using lipofectamine PLUS (Invitrogen) following the manufacturer's protocol. Cells were harvested 5 days post-transfection when EGFP-positive plaques were observed. Recombinant viruses, identified as EGFP-positive plaques, were plaque purified on E5 cells by three rounds of limiting dilution.

### Restriction analysis of viral DNA

Viral DNA for restriction analysis was purified as previously described with some modifications [50]. Briefly, E5 cells plated in 10 cm culture dish was infected with virus and harvested when total CPE was observed. The cells were pelleted by centrifugation, resuspended in buffer (10 mM TrisHCl pH7.5, 10 mM NaCl, 3 mM MgCl_2_) and lysed by three freeze-thaw cycles. After centrifugation at 2000 *g *4°C for 10 min, the supernatant was collected and treated with RNase A (QIAGEN) and DNase I (Sigma, St. Louis, MO) at 37°C for 2 hours, and subsequently treated with Proteinase K and SDS at 56°C overnight. Viral DNA was purified by phenol-chloroform extraction and ethanol precipitation. For restriction analysis, viral and BAC DNA was digested with Hind III and separated by electrophoresis in 0.5% agarose/Tris-acetate-EDTA buffer. A 1-kb DNA ladder (Invitrogen) and a high-molecular-weight DNA marker (Invitrogen) were used as molecular weight standards.

### Cloning of a BAC clone containing the whole genome of M24-BAC virus

Isolation of a BAC clone containing the whole genome of M24-BAC virus was performed as reported previously [14]. Briefly, Vero cells (6 × 10^5 ^cells/well) plated in six-well dishes were infected with M24-BAC at an MOI of 3. Cells were lysed 90 min after infection with 500 μl of lysis buffer (0.6% SDS, 10 mM EDTA pH 7.5) and incubated at room temperature for 20 min. Then 330 μl of 5 M NaCl was added to the lysate and the mixture was incubated on ice for 5 hours. After centrifugation at 16000 *g *for 20 min at 4°C, the supernatant (Hirt extract) was removed, DNA extracted with phenol/chloroform, ethanol precipitated, and dissolved in 5 μl of TE buffer. Electrocompetent DH10B cells (Invitrogen) were transformed with 2 μl of DNA using Gene Pulser II (Bio-Rad, Hercules, CA) and chloramphenicol resistant clones were isolated.

### Integration of shuttle vectors with pM24-BAC by Cre recombinase

Site-specific recombination between pM24-BAC and shuttle vector DNA was performed as previously described with some modifications [29]. Briefly, 1 to 1.5 μg of pM24-BAC and 150 ng of shuttle vector DNA were mixed with Cre recombinase (Invitrogen) in a total volume of 10 μl and incubated at 37°C for 1 h. Following heat-inactivation of Cre recombinase at 70°C for 10 min, recombined DNA was ethanol precipitated and dissolved in 5 μl of TE buffer or dH2O. Electrocompetent DH10B cells were transformed with 1 μl of DNA using Gene Pulser II and chloramphenicol/kanamycin double-resistant clones were isolated.

### Recombinant virus production from integrated pM24-BAC-shuttle DNA

E5 cells (1.5 × 10^5 ^cells/well) plated in 24-well dishes were co-transfected with 1 μg of pM24-BAC-shuttle DNA and 100 ng of pCAGGS-FLPe using lipofectamine PLUS following manufacturer's protocol. 5 to 7 days after transfection, when CPE is observed, cells were harvested and lysed by three freeze/thaw cycles and sonication. Recombinant viruses were plaque purified on E5 cells by limiting dilution.

### Virus replication

Monolayer cultures of cells in 12-well dishes were infected with the designated virus at an MOI of 3 in 350 μl of DMEM with 1%IFCS (for single step growth) or cells in 6-well dishes were infected with 1 × 10^4 ^pfu of the designated virus (MOI of ~0.01) in 0.7 ml of DMEM with 1%IFCS (for replication assay). The virus inoculum was removed after 2 hours and the cells were incubated in DMEM supplemented with 10% IFCS (or 1% IFCS for Vero cells) at 37°C in humidified 5% CO_2_. At the times indicated, virus was harvested from the wells and titers were determined on Vero cells (for KOS and *hr*R3) or on E5 cells (for other recombinant viruses).

### Immunoblot analysis of ICP4

Monolayer cultures of cells grown in 12-well dishes were infected with the designated virus at an MOI of 2, as described above. At the times indicated, the cells were briefly washed with PBS and lysed with 1 × SDS sample buffer. Proteins obtained from ~3 × 10^4 ^infected cells were separated by SDS-PAGE (5% gel) and transferred to PVDF membrane (Immobilon-P, Millipore, Bedford, MA) using a tank transfer system (Bio-Rad). Membranes were blocked with 5% non-fat dry milk in TBST (10 mM Tris pH8, 150 mM NaCl, 0.1% Tween-20), washed in TBST, and probed with mouse anti-HSV-ICP4 monoclonal antibody (US Biological, Swampscott, MA) diluted in TBST (1:6000 dilution). After washing in TBST, the blot was probed with HRP-linked anti-mouse-IgG antibody (Amersham Biosciences, Piscataway, NJ). The membranes were washed in TBST and chemiluminescent detection was performed using the ECL Western Blotting Detection Reagents (Amersham Biosciences). Molecular weights were determined using prestained protein standards (Bio-Rad).

## Authors' contributions

TK participated in the design of the study, designed and constructed the vectors, carried out molecular biological and virological experiments, and drafted the manuscript. TT participated in the design of the experiments; RLM and SR participated in the design and interpretation of the experiments, and in drafting the manuscript. All the authors read and approved the final manuscript.

## Supplementary Material

Additional File 1 **Genomic structure of M24-BAC and bM24-BAC viral DNA**. HindIII restriction analysis of purified viral DNAs obtained from M24-BAC and bM24-BAC virus isolates #1 and #2, which are identical. Click here for file

Additional File 2 **Replication of M24-BAC and bM24-BAC in E5 cells**. (**a**) Replication assay of recombinant viruses. 3.8 × 10^5 ^E5 cells grown in 12-well plates were infected with the indicated viruses at an MOI of 0.02, viruses were harvested at the indicated times and titered on E5 cells. (**b**) Virus yield of indicated viruses 74 hours after infection of E5 cells at an MOI of 0.02, from the same experiment as in a. (Error bars show standard deviation. N = 3) Click here for file

Additional File 3 **Schematic Hind III restriction map of the UL39 region in the recombinant viruses and BAC plasmids**. The numbers in red show the lengths in kb of HindIII restriction fragments. H, HindIII. Click here for file

## Figures and Tables

**Table 1:** List of recombinant viruses and shuttle and BAC plasmid clones used for their generation

BAC-derived recombinant virus	ICP4 expression cassette on the vector	Shuttle vector	Integrated pM24-BAC-shuttle clone	Description
bM24-CMV		pFLS-CMV	pM24-BAC-CMV	The CMV promoter drives the ICP4 expression.
bM24-null		pFLS-ICP4	pM24-BAC-null	No active promoter drives the ICP4 expression.
bM24-empty	NONE	pFLS2	pM24-BAC-empty	No ICP4 CDS included on the vector. (A shuttle vector containing no ICP4 CDS was constructed)

bM24-BAC	N/A	N/A	pM24-BAC (Non-integrated prototype HSV-BAC)	Genetically identical to the prototype HSV-BAC virus M24-BAC

(N/A: not applicable)
