# Supplementary figures and images for: Flip-Flop HSV-BAC: bacterial artificial chromosome based system for rapid generation of recombinant herpes simplex virus vectors using two independent site-specific recombinases
Source: BMC Biotechnol. 2006 Sep 22;6:40. doi: 10.1186/1472-6750-6-40 (PMC1609115; doi:10.1186/1472-6750-6-40)

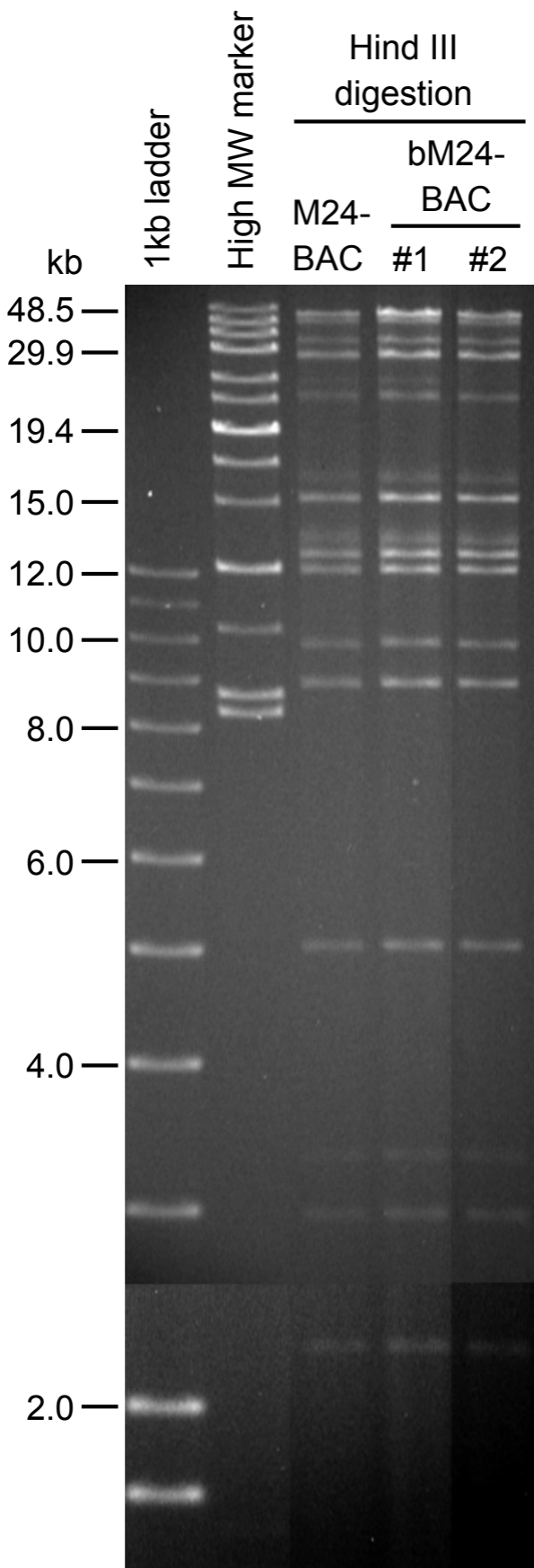

Supplement: Additional File 1 — Genomic structure of M24-BAC and bM24-BAC viral DNA. HindIII restriction analysis of purified viral DNAs obtained from M24-BAC and bM24-BAC virus isolates #1 and #2, which are identical. [file 1472-6750-6-40-S1.pdf]

a

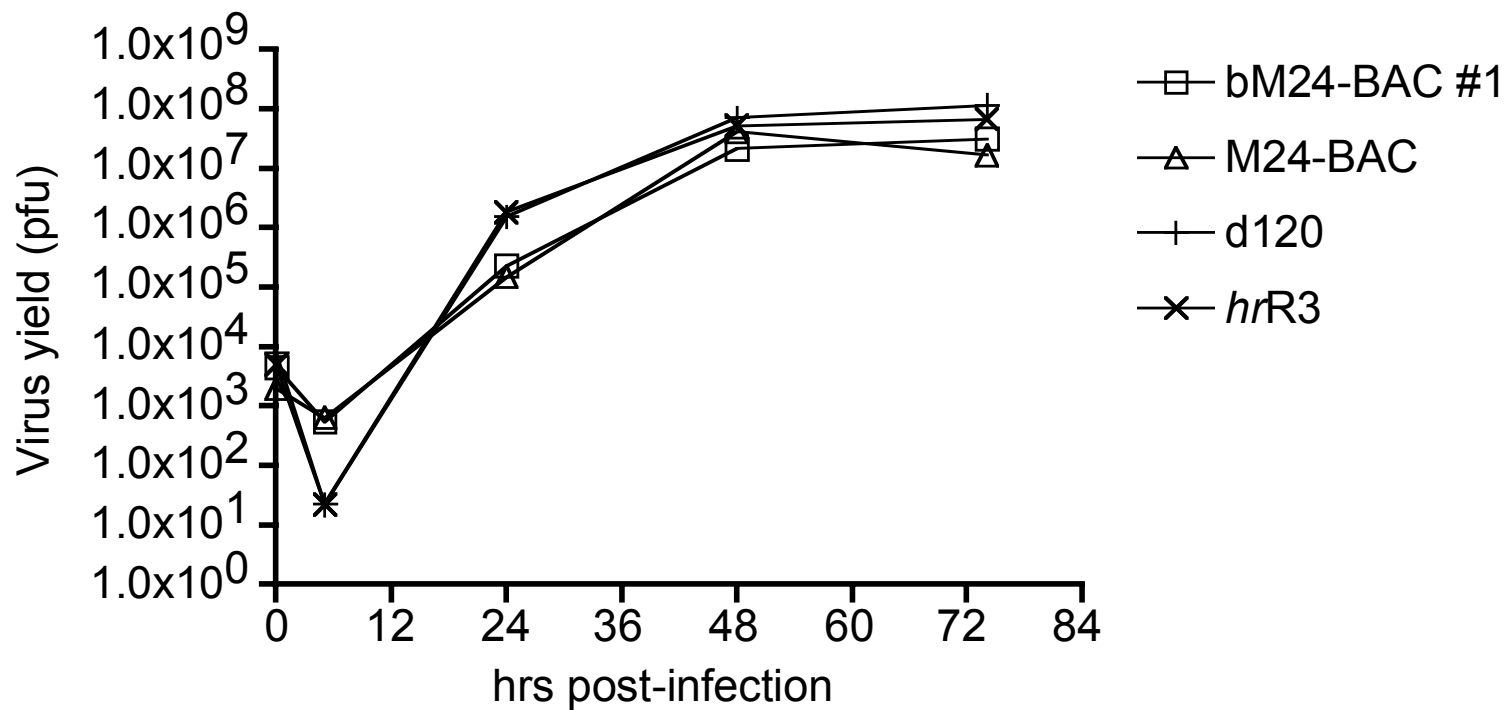

b

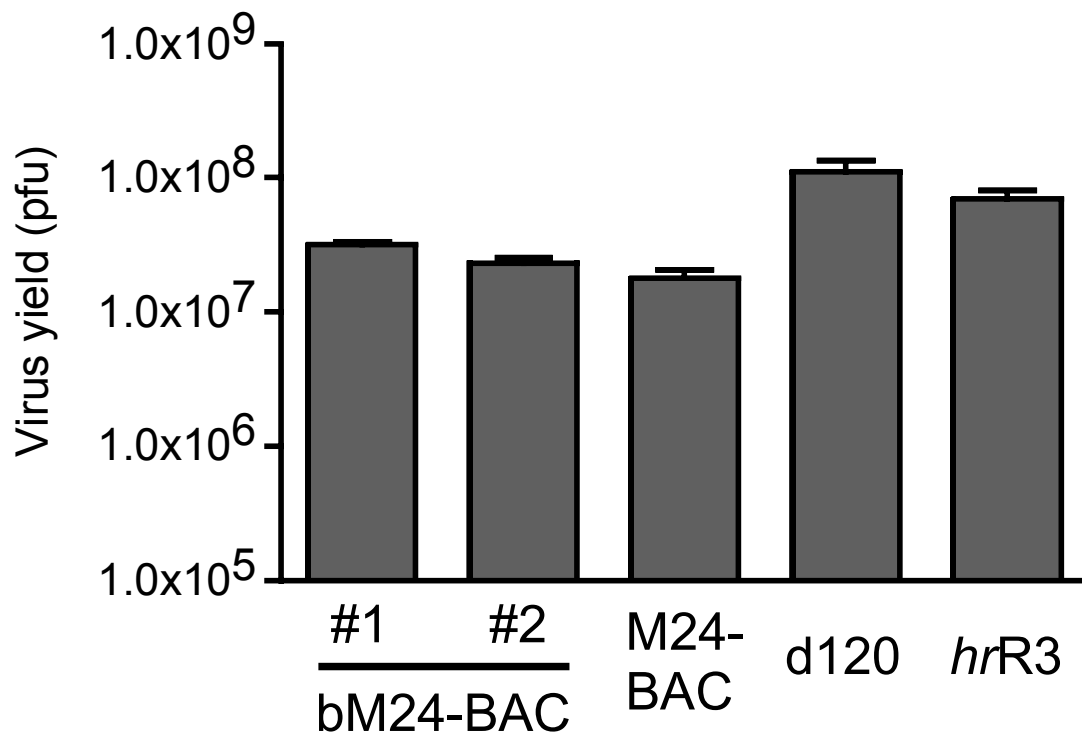

Supplement: Additional File 2 — Replication of M24-BAC and bM24-BAC in E5 cells. (a) Replication assay of recombinant viruses. 3.8 × 10^5^ E5 cells grown in 12-well plates were infected with the indicated viruses at an MOI of 0.02, viruses were harvested at the indicated times and titered on E5 cells. (b) Virus yield of indicated viruses 74 hours after infection of E5 cells at an MOI of 0.02, from the same experiment as in a. (Error bars show standard deviation. N = 3) [file 1472-6750-6-40-S2.pdf]
