# Supplementary material for: Flip-Flop HSV-BAC: bacterial artificial chromosome based system for rapid generation of recombinant herpes simplex virus vectors using two independent site-specific recombinases
Source: BMC Biotechnol. 2006 Sep 22;6:40. doi: 10.1186/1472-6750-6-40 (PMC1609115; doi:10.1186/1472-6750-6-40)
Supplement: Additional File 3 — Schematic Hind III restriction map of the UL39 region in the recombinant viruses and BAC plasmids. The numbers in red show the lengths in kb of HindIII restriction fragments. H, HindIII. [file 1472-6750-6-40-S3.pdf]

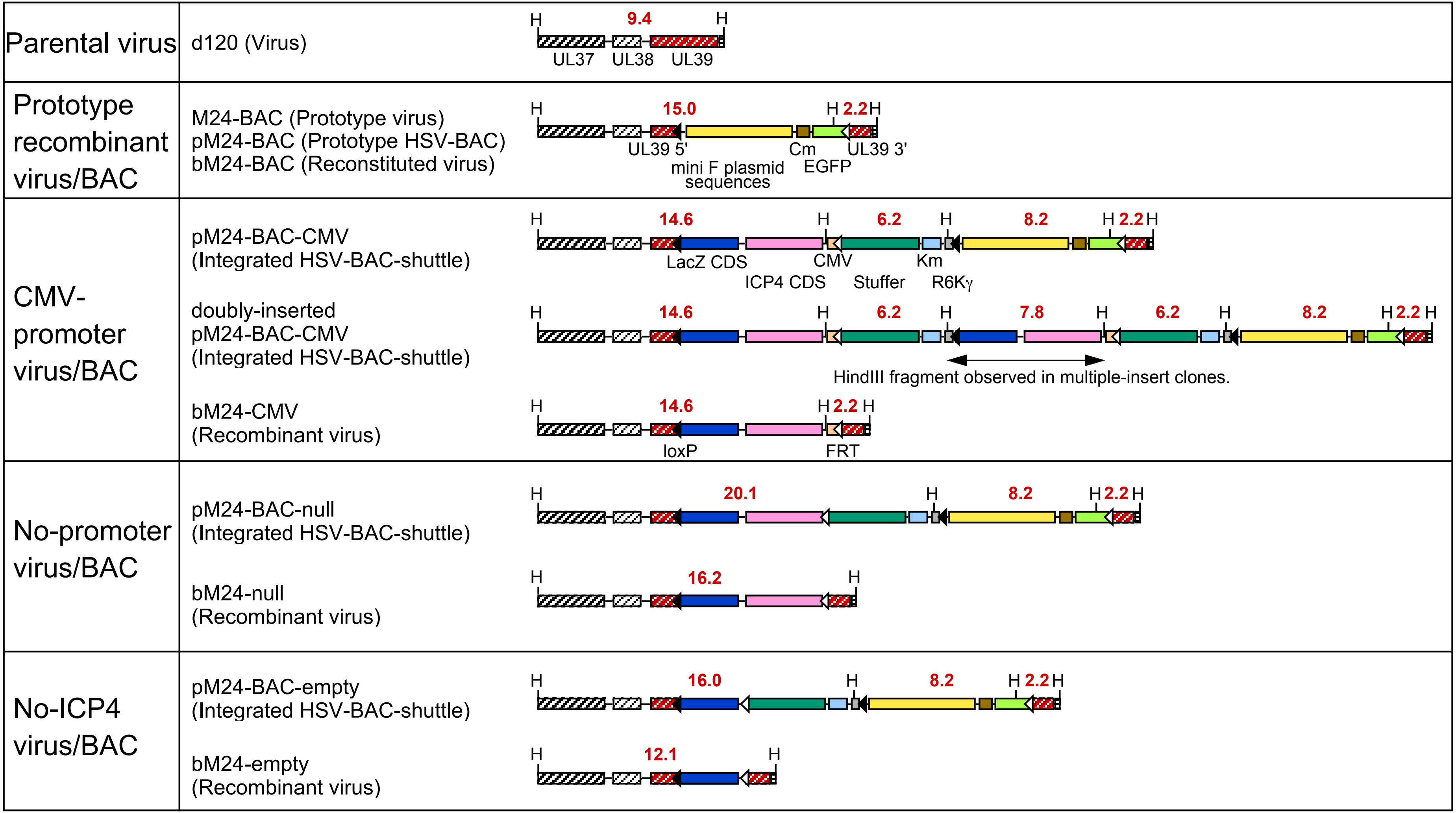

Parental virus
d120 (Virus)
H 9.4 H
UL37 UL38 UL39
Prototype recombinant virus/BAC
M24-BAC (Prototype virus)
pM24-BAC (Prototype HSV-BAC)
bM24-BAC (Reconstituted virus)
H 15.0 H 2.2 H
UL39 5'
mini F plasmid sequences
Cm
EGFP
UL39 3'
CMV-promoter virus/BAC
pM24-BAC-CMV
(Integrated HSV-BAC-shuttle)
H 14.6 H 6.2 H 8.2 H 2.2 H
LacZ CDS
ICP4 CDS
CMV
Stuffer
Km
R6Kγ
doubly-inserted pM24-BAC-CMV
(Integrated HSV-BAC-shuttle)
H 14.6 H 6.2 H 7.8 H 6.2 H 8.2 H 2.2 H
HindIII fragment observed in multiple-insert clones.
bM24-CMV
(Recombinant virus)
H 14.6 H 2.2 H
loxP
FRT
No-promoter virus/BAC
pM24-BAC-null
(Integrated HSV-BAC-shuttle)
H 20.1 H 8.2 H 2.2 H
bM24-null
(Recombinant virus)
H 16.2 H
No-ICP4 virus/BAC
pM24-BAC-empty
(Integrated HSV-BAC-shuttle)
H 16.0 H 8.2 H 2.2 H
bM24-empty
(Recombinant virus)
H 12.1 H
